# Preclinical evaluation of Ayurveda and Siddha Herbo-mineral formulations in cancer: A scoping review protocol

**DOI:** 10.1371/journal.pone.0355873

**Published:** 2026-08-14

**Authors:** Anjali Saini, Shalini Rai

**Affiliations:** All India Institute of Ayurveda, New Delhi, India; Dr Anjali Chatterji Regional Research Institute for Homoeopathy, INDIA

## Abstract

**Background:**

Cancer poses a major global health challenge, with rising incidence and mortality rates straining healthcare systems worldwide. Despite remarkable advances in cancer management modalities, the current approaches still fall short in overcoming drug resistance, tumour heterogeneity, toxicity and side effects. Traditional systems such as Ayurveda and Siddha may help overcome these limitations of conventional medicine; however, robust evidence supporting the anticancer efficacy of Herbo mineral formulations (HMFs) remains limited and fragmented.

**Objective:**

This scoping review is planned to map the existing pre-clinical evidence from in vitro and in vivo studies for the anticancer activities of Ayurveda and Siddha HMFs.

**Methods:**

The scoping review will be conducted in accordance with the framework proposed by Arksey and O’Malley and the recommendations of the Joanna Briggs Institute for conducting scoping reviews. A systematic search will be conducted by two independent reviewers across selected databases for relevant literature, and studies will be selected on the basis of predefined eligibility criteria. Data from the included studies will be presented in tabular and narrative formats to align with the review objectives. The review will be reported in accordance with the Preferred Reporting Items for Systematic Reviews and Meta-Analyses extension for Scoping Reviews guidelines.

**Discussion:**

This review will contribute to integrating traditional medicine with modern oncology, guiding researchers and clinicians in prioritizing promising anticancer drugs for further investigation and potential therapeutic use. The review protocol was registered with the Open Science Framework (OSF) and is available at: osf.io/rdhpv/.

## Introduction

The global cancer burden is substantial and rising, and it is projected to continue increasing through 2050, with the disproportionate growth expected in lower-resource regions. [1] The current global statistics for 2022 report approximately 20 million new cancer diagnoses and nearly 10 million cancer-related deaths worldwide. The estimates indicate that nearly one in five men or women develop cancer in a lifetime, whereas around one in nine men and one in 12 women die from it. Demographic projections indicate that annual cancer incidence will reach about 35 million cases by 2050, representing a 77% increase from 2022 levels. [2]

Conventional cancer treatments, including surgery, radiotherapy, chemotherapy, and hormonal therapy, have seen significant advances in both effectiveness and precision. Despite these advances, cancer therapy still faces several major challenges. Chemotherapy and hormone therapy are often limited by resistance caused by genetic mutations, altered signalling pathways, and tumour heterogeneity. [3] These limitations and the ongoing rise in cancer-related deaths underscore the urgent need for alternative therapies. Traditional systems of medicine could help overcome these limitations of conventional medicine, either as standalone therapies or as adjuvants.

Drugs used in traditional systems of medicine are diverse as they might be purely herbal, Herbo-mineral or animal-based. Herbo-mineral formulations (HMFs) are pharmaceutical preparations composed of herbal ingredients combined with purified and processed minerals and/or metals, prepared through classical procedures such as *Shodhana* (purification) for detoxification and *Marana* (calcination/incineration) to achieve enhanced therapeutic efficacy. [4,5] HMFs are utilized in various traditional systems of medicine, such as Ayurveda, Siddha, Unani, Traditional Chinese medicine, and Sowa Rigpa (Tibetan medicine). However, this review focuses on Ayurveda and Siddha, which extensively use HMFs and share similar metal-processing techniques in their preparations.

Metallic nanoparticles are emerging as a novel strategy for targeted drug delivery systems and cancer diagnosis. [6] Metals currently employed in nanomedicine include iron, zinc, copper, and noble metals such as gold, silver, and platinum. Recent preclinical studies and early clinical trials suggest that they hold promise for cancer treatment. However, their tendency to accumulate and resist clearance in non-malignant tissues can lead to oxidative stress, metabolic disturbances, macromolecular dysfunction, and ultimately cell death. [7] Green synthesis of nanomedicine offers a cost-effective and eco-friendly alternative to conventional chemical and physical methods. Ayurvedic *Bhasma* (Herbo-mineral-metallic compounds having mixture of micro or nano particles) can be viewed as analogous to green-synthesized nanoparticles in modern science. The use of plant extracts enhances biocompatibility, stability, safety, and therapeutic potential, making such formulations more effective and environmentally sustainable. [8]

HMFs, described in Ayurveda as *Rasaushadhi,* have significant advantages over herbal preparations, including smaller therapeutic doses, quicker action, enhanced potency, and a longer shelf life. However, herbal medicines are more widely accepted and have received the attention of researchers across the globe. Evidence regarding the efficacy and safety of HMFs is still limited.

A preliminary literature search identified no published scoping or systematic reviews on the preclinical anticancer effects of HMFs. In vitro and in vivo studies are essential preliminary steps for the scientific validation of the anticancer efficacy and safety of traditional drugs. However, the existing experimental evidence on Ayurveda and Siddha HMFs in cancer is scattered across diverse cancer models, formulations, study designs, and outcome measures, limiting a comprehensive assessment of their therapeutic potential and research gaps. This scoping review aims to systematically map the available evidence, categorize the formulations and experimental approaches studied, and identify knowledge gaps to inform future research and standardization efforts in cancer pharmacology.

### Research question

What pre-clinical evidence exists from in vitro and in vivo studies regarding the anticancer activities of Ayurveda and Siddha HMFs?

### Objectives

The objectives of this scoping review are to:

- Identify specific Ayurveda and Siddha HMFs that have been evaluated for anticancer activity through in vitro and in vivo studies.
- Assess the cancer models (cell lines or animal models) and study designs employed in these studies.
- Summarize the reported anticancer outcomes and proposed mechanisms of action (if any).
- Identify knowledge gaps and limitations in the existing preclinical evidence.

## Methods

### Study design

This scoping review protocol is developed following the framework proposed by Arksey and O’Malley [9] and adheres to the recommendations of the Joanna Briggs Institute (JBI) [10] for conducting scoping reviews. The protocol is reported in accordance with the Preferred Reporting Items for Systematic Reviews and Meta-Analyses extension for Scoping Reviews (PRISMA-ScR) guidelines. [11] This protocol has been registered in Open science frameworkavailable at: osf.io/rdhpv/).

### Search strategy

The scoping review will focus on three key concepts: (i) cancer cell line or animal model, (ii) Anticancer activities of Ayurveda and Siddha HMFs, and (iii) Experimental studies (in vitro and in vivo), following the Population–Concept–Context (PCC) framework recommended by the Joanna Briggs Institute (JBI). PubMed/MEDLINE, Scopus and Google Scholar will be searched systematically for relevant literature. PubMed is selected for its comprehensive biomedical coverage, Scopus for its broad multidisciplinary indexing, and Google Scholar for identifying additional studies that may not be indexed in PubMed or Scopus. These databases provide sufficient coverage of preclinical pharmacology and oncology research on traditional HMFs. Grey literature sources, including theses and dissertations, will be excluded to ensure consistency in methodological and reporting standards across included studies.

A three-step search strategy will be employed to ensure comprehensive coverage. An initial limited search of PubMed will identify relevant articles and refine keywords and subject headings. Then, a comprehensive search will be conducted across Scopus and Google Scholar using the refined terms combined with Boolean operators (“AND,” “OR,” “NOT”) to capture all pertinent studies. Additionally, reference lists of included studies, will be screened to identify further relevant articles.

Initial search strategy for PubMed is provided below:

“Herbo mineral” [Title/Abstract] OR “Herbo-mineral” [Title/Abstract] OR herbomineral[Title/Abstract] OR “Herbo metallic” [Title/Abstract] OR “Herbo-metallic” [Title/Abstract] OR herbometallic[Title/Abstract] OR Bhasma[Title/Abstract] OR rasaushadhi[Title/Abstract] OR Siddha[Title/Abstract]

AND

“Neoplasms”[Mesh] OR Cancer[Title/Abstract] OR neoplasm*[Title/Abstract] OR tumor[Title/Abstract] OR tumour[Title/Abstract] OR malignan*[Title/Abstract] OR carcinoma*[Title/Abstract] OR sarcoma*[Title/Abstract] OR leukemia[Title/Abstract] OR leukaemia[Title/Abstract] OR lymphoma[Title/Abstract] OR anticancer[Title/Abstract] OR “anti-cancer” [Title/Abstract] OR antitumor[Title/Abstract] OR “anti-tumor” [Title/Abstract] OR antineoplastic[Title/Abstract]

AND

“Drug Evaluation, Preclinical”[Mesh] OR Preclinical[Title/Abstract] OR “Animal Experimentation”[Mesh] OR “Neoplasms, Experimental”[Mesh] OR “in vivo” [Title/Abstract] OR “in-vivo” [Title/Abstract] OR “invivo” [Title/Abstract] OR animal[Title/Abstract] OR murine[Title/Abstract] OR mouse*[Title/Abstract] OR mice[Title/Abstract] OR rat*[Title/Abstract] OR xenograft*[Title/Abstract] OR tumor-bearing[Title/Abstract] OR tumour-bearing[Title/Abstract] OR “Cell Line, Tumor”[Mesh] OR “In Vitro Techniques”[Mesh] OR “in vitro” [Title/Abstract] OR “in-vitro” [Title/Abstract] OR “invitro” [Title/Abstract] OR “cell line*”[Title/Abstract] OR “cell culture*”[Title/Abstract] OR “cultured cells” [Title/Abstract] OR “cancer cell*”[Title/Abstract] OR “tumor cell*”[Title/Abstract] OR “tumour cell*”[Title/Abstract]

Full database-specific search strings are provided in the supplementary file S1 Appendix.

### Eligibility criteria

#### Inclusion criteria.

The in vitro and in vivo experimental studies evaluating anticancer activity of HMFs of Ayurveda and Siddha system will be included for review. No restrictions will be placed on the year and language of publication to allow comprehensive mapping of the available evidence. Studies will be included only if their full-text articles are accessible.

#### Exclusion criteria.

Studies will be excluded if

- they involve clinical studies involving human participants, or computational and in silico models without in vitro or in vivo experimental component.
- Review articles, book chapters, editorials, conference abstracts, study protocols, theses, and dissertations.
- In vitro or in vivo studies not related to cancer.
- Studies assessing purely herbal drugs or metallic drugs from systems other than Ayurveda and Siddha, in accordance with the predefined scope of the review.
- Laboratory-synthesized metals and nanoparticles, including Nanomedicine studies inspired by traditional medicine but prepared using modern synthesis methods without classical processes (*Shodhana, Bhavana, Maraṇa*), will be excluded. These interventions differ fundamentally from traditional HMFs in their preparation methods, conceptual basis, and regulatory framework, with therapeutic activity primarily determined by engineered nanoscale properties.

### Selection of sources of evidence

All identified records will be imported into Zotero 7.0.30 and duplicates will be removed. Two reviewers will independently screen titles and abstracts against the predefined inclusion and exclusion criteria using Rayyan. Articles that meet the initial screening criteria will undergo full-text review to determine eligibility. Any disagreements between reviewers will be resolved through discussion between the reviewers. If consensus cannot be reached, an independent third reviewer will be consulted to adjudicate the disagreement and make the final decision. A PRISMA-ScR flow diagram will be used to document the selection process, including the number of records identified, screened, excluded, and included in the scoping review.

### Data extraction and charting

Data will be extracted from papers included in the scoping review by two independent reviewers using a data charting form (S2 Appendix) developed specifically for this scoping review. For in vitro studies, extracted information will include bibliographic details, HMFs characteristics (e.g., name, composition, and medicine system), cancer type and cell line, culture conditions, intervention and control groups, dose and treatment duration, assays employed, mechanisms or pathways investigated, safety related findings, key results, and study limitations. For in vivo studies, extracted information will include bibliographic details, HMFs characteristics, animal characteristics, cancer model type, intervention and control groups, randomization and blinding (if reported), dose and route of administration, outcome measures, mechanisms investigated, toxicity findings, ethical compliance, key results, and study limitations.

Consistent with scoping review methodology, a formal critical appraisal will not be conducted. However, variations in study design, methodological characteristics, and potential limitations will be documented descriptively during data charting and while presenting the findings. Extracted data will be arranged in a Microsoft Excel sheet. A pilot data extraction will be performed on five selected studies, and the form will be refined as necessary. Any disagreements that arise between the reviewers will be resolved through discussion or consultation with an independent third reviewer.

The draft data extraction tool will be modified and revised as necessary during the process of extracting data from each included evidence source. Modifications will be detailed in the scoping review.

### Data analysis and presentation

Following data extraction, the included studies will be collated, summarized, and synthesized descriptively. Studies will first be categorized as in vitro or in vivo. Within each category, studies will be further grouped according to the origin of the HMFs (e.g., Ayurveda or Siddha) and the cancer type or model investigated. A descriptive numerical summary of study characteristics will be presented alongside a narrative synthesis to map key concepts, including HMFs characteristics, cancer models, efficacy outcomes, mechanisms of action, safety findings, and reported limitations.

The study selection process will be presented using a PRISMA-ScR flow diagram, and the findings will be presented in tabular and narrative formats to align the results with the review objectives and to identify research gaps in the existing literature.

### Protocol amendments

Scoping reviews are iterative by nature. Any significant amendments will be documented with the date, description, and rationale, and reported in the registry and final review.

### Ethical clearance

This study will use secondary data from publicly available databases; therefore, ethical approval is not required.

## Discussion

The discovery and development of novel drugs originating from natural products have long been a focus of researchers. [12] Majority (>60%) of anticancer drugs used clinically are derived from natural sources such as plants, marine organisms and micro-organisms. [13] The journey of an entirely new drug from laboratory to market approval is a resource-intensive process. Traditional systems of medicine may offer promising therapeutic options for diseases like cancer. By leveraging reverse pharmacology, they circumvent the conventional drug discovery approach.

Over recent years, metal-based nanomaterials have been widely studied for their potential applications in drug delivery systems, especially in the treatment of cancers. Metal nanoparticles offer a model to achieve the main objectives of drug delivery like targeted therapy, minimizing side effects, and controlled drug release. [14] Metal nanoparticles have high drug loading, multifunctionality, and good cellular uptake. However, their use is limited by critical concerns, including potential cytotoxicity, immunogenic responses, inflammation, oxidative stress, long-term accumulation in organs, upscaling from laboratory to industry, high cost of premium-grade metals, or inadequate regulatory frameworks. Consequently, despite encouraging preclinical results, metal nanoparticles often fail in Phase II and Phase III clinical trials. [15]

Traditional HMFs share certain physicochemical features with metal nanoparticles, including nano/submicron particle dimensions and high surface-area-to-volume ratios, as demonstrated by modern characterization studies of several *Bhasmas*. [16,17] However, HMFs are complex formulations produced through multistep purification and calcination processes and therefore differ from engineered or green-synthesized nanoparticles in composition, surface chemistry, and biological interactions. [8] Nonetheless, HMFs have been used in clinics for centuries to treat a wide range of diseases. Owing to their well-established empirical and clinical foundation, HMFs may offer greater translational success potential.

Nevertheless, traditional medicines are not devoid of safety concerns. Traditional medicines are often perceived as inherently safe and free from adverse effects. However, contrary to this common belief, HMFs present significant safety concerns due to the presence of potentially toxic heavy metals, including lead, mercury, and arsenic. Excessive exposure to these metals may result in cumulative toxicity affecting the nervous, renal, hepatic, and hematopoietic systems. [18,19] Cases of metal toxicity associated with HMFs are primarily attributed to inappropriate use, including excessive doses and prolonged administration, as well as improper preparation procedures. [20] Furthermore, contamination, adulteration, and inadequate quality assurance during production may increase the risk of heavy-metal exposure. Such reports have highlighted the need for stringent quality control and robust post-marketing surveillance. However, ensuring the safety of HMFs remains challenging due to variability in manufacturing practices, difficulties in standardization, and inconsistencies in regulatory frameworks governing traditional medicines. [18,21] In India, Ayurvedic, Siddha, and Unani medicines are regulated by the Ministry of Ayush under the Drugs and Cosmetics Act, with pharmacovigilance initiatives established to monitor adverse drug reactions and enhance patient safety. [22] The World Health Organization (WHO) also advocates the inclusion of traditional medicines into pharmacovigilance systems and provides guidelines for their safety monitoring. [23] Therefore, the safe use of HMFs requires stringent raw-material quality control, compliance with Good Manufacturing Practices (GMP), validated detoxification and processing methods, and continuous pharmacovigilance to ensure that toxic metal levels remain within acceptable limits and do not pose undue health risks.

HMFs are classically claimed to be more potent, with longer shelf life, effective in smaller doses and faster acting in comparison to purely herbal drugs. But the scientific evidence regarding the safety, anticancer efficacy and mechanistic studies of HMFs remains insufficient.

This scoping review aims to map the existing preclinical evidence of anticancer effects of HMFs. This review will provide a comprehensive overview of investigated traditional mineral-based drugs, targeted cancer type, experimental models, dosage, and any reported mechanisms of action.

Additionally, this review will highlight the research gap and limitations of the existing studies. A preliminary search across databases indicates that research on the use of HMFs in cancer is confined to only laboratory studies and case reports. Identifying the challenges in translating preclinical research into clinical trials will help shape the work of future researchers. The findings of this review will contribute to bridging the traditional medicine and modern oncology by providing a structured summary of existing evidence. This can guide researchers and clinicians in prioritizing promising drugs for further investigation and potential therapeutic use.

This review is limited to preclinical studies, such as in vitro and animal experiments, because randomized clinical trials are not yet available. Preclinical studies vary widely in quality and methodologies. This scoping review aims to map the available evidence rather than critically appraising study validity in detail. Additionally, the review may be limited by variability in the terminology used to describe HMFs and exclusion of studies on laboratory-synthesized metals and nanoparticles. Despite these limitations, the scoping review approach is well suited to provide the initial groundwork for subsequent research on traditional HMFs.

## Supporting information

S1 Appendix Search strategy. (DOCX)

S2 Appendix Draft data extraction instrument. (DOCX)

S3 Appendix PRISMA-P (Preferred Reporting Items for Systematic review and Meta-Analysis Protocols) 2015 checklist. (DOCX)

## Abbreviations

HMFs: Herbomineral formulations
PRISMA-ScR: Preferred Reporting Items for Systematic Reviews and Meta-Analyses extension for Scoping Reviews
