# Supplementary material for: Preclinical evaluation of Ayurveda and Siddha Herbo-mineral formulations in cancer: A scoping review protocol
Source: PLoS One. 2026 Aug 14;21(8):e0355873. doi: 10.1371/journal.pone.0355873 (PMC13475941; doi:10.1371/journal.pone.0355873)
Supplement: Supplementary file 1 [file pone.0355873.s001.docx]

**Preclinical evaluation of Ayurveda and Siddha Herbo-mineral formulations in cancer: A scoping review protocol**

Anjali Saini^1^, Shalini Rai^1*^

1. All India Institute of Ayurveda, New Delhi, Delhi, India.

* Corresponding author e-mail: [vd.raishalini@gmail.com](mailto:vd.raishalini@gmail.com) (SR)

## S1 Appendix: Search strategy

Search strategy for PubMed (Date of search: 8^th^ Jan 2026)

| Search | Query | Records retrieved |
| --- | --- | --- |
| 1. | “Herbo mineral” [Title/Abstract] OR “Herbo-mineral” [Title/Abstract] OR herbomineral[Title/Abstract] OR “Herbo metallic” [Title/Abstract] OR “Herbo-metallic” [Title/Abstract] OR herbometallic[Title/Abstract] OR Bhasma[Title/Abstract] OR rasaushadhi[Title/Abstract] OR Siddha[Title/Abstract] | 1,537 |
| 2. | "Neoplasms"[Mesh] OR Cancer[Title/Abstract] OR neoplasm*[Title/Abstract] OR tumor[Title/Abstract] OR tumour[Title/Abstract] OR malignan*[Title/Abstract] OR carcinoma*[Title/Abstract] OR sarcoma*[Title/Abstract] OR leukemia[Title/Abstract] OR leukaemia[Title/Abstract] OR lymphoma[Title/Abstract] OR anticancer[Title/Abstract] OR “anti-cancer” [Title/Abstract] OR antitumor[Title/Abstract] OR “anti-tumor” [Title/Abstract] OR antineoplastic[Title/Abstract] | 5,729,729 |
| 3. | "Drug Evaluation, Preclinical"[Mesh] OR Preclinical[Title/Abstract] OR "Animal Experimentation"[Mesh] OR "Neoplasms, Experimental"[Mesh] OR “in vivo” [Title/Abstract] OR “in-vivo” [Title/Abstract] OR “invivo” [Title/Abstract] OR animal[Title/Abstract] OR murine[Title/Abstract] OR mouse*[Title/Abstract] OR mice[Title/Abstract] OR rat*[Title/Abstract] OR xenograft*[Title/Abstract] OR tumor-bearing[Title/Abstract] OR tumour-bearing[Title/Abstract] OR "Cell Line, Tumor"[Mesh] OR "In Vitro Techniques"[Mesh] OR “in vitro” [Title/Abstract] OR “in-vitro” [Title/Abstract] OR “invitro” [Title/Abstract] OR “cell line*”[Title/Abstract] OR “cell culture*”[Title/Abstract] OR “cultured cells” [Title/Abstract] OR “cancer cell*”[Title/Abstract] OR “tumor cell*”[Title/Abstract] OR “tumour cell*”[Title/Abstract] | 6,060,978 |
| 4. | #1 AND #2 AND #3 | 109 |

Search strategy for Scopus (Date of search: 12^th^ Feb 2026)

TITLE-ABS-KEY ( ( "Herbo mineral" OR "Herbo-mineral" OR herbomineral OR "Herbo metallic" OR "Herbo-metallic" OR herbometallic OR Bhasma OR rasaushadhi OR Siddha OR Unani ) AND ( Cancer OR neoplasm* OR tumor OR tumour OR malignan* OR carcinoma* OR sarcoma* OR leukemia OR leukaemia OR lymphoma OR anticancer OR "anti-cancer" OR antitumor OR "anti-tumor" OR antineoplastic ) AND ( Preclinical OR "in vivo" OR "in-vivo" OR "invivo" OR animal OR murine OR mouse* OR mice OR rat* OR xenograft* OR tumor-bearing OR tumour-bearing OR "in vitro" OR "in-vitro" OR "invitro" OR "cell line*" OR "cell culture*" OR "cultured cells" OR "cancer cell*" OR "tumor cell*" OR "tumour cell*" ) ) AND ( LIMIT-TO ( DOCTYPE , "ar" ) )

229 results retrieved.

Search strategy for Google Scholar (last date of search: 25^th^ Feb 2026)

(Herbo mineral OR Herbo metallic OR Bhasma OR Rasaushadhi OR Siddha OR Unani) AND (Cancer OR neoplasm OR tumour OR carcinoma OR leukaemia) AND (Preclinical OR in vivo OR animal OR rat OR in vitro OR “cell line” OR “cancer cell”)

1170 results retrieved.
