# Supplementary material for: Preclinical evaluation of Ayurveda and Siddha Herbo-mineral formulations in cancer: A scoping review protocol
Source: PLoS One. 2026 Aug 14;21(8):e0355873. doi: 10.1371/journal.pone.0355873 (PMC13475941; doi:10.1371/journal.pone.0355873)
Supplement: Supplementary file 2 [file pone.0355873.s002.docx]

**Preclinical evaluation of Ayurveda and Siddha Herbo-mineral formulations in cancer: A scoping review protocol**

Anjali Saini^1^, Shalini Rai^1*^

1. All India Institute of Ayurveda, New Delhi, Delhi, India.

* Corresponding author e-mail: [vd.raishalini@gmail.com](mailto:vd.raishalini@gmail.com) (SR)

## S2 Appendix: Draft data extraction instrument

The following items will be assessed during data extraction:

## For in vitro studies

- Bibliographic details (author, year)
- Drug/Formulation (name, composition, medicine system)
- Cell line / Cancer type / Cell source
- Culture Conditions
- Groups / Controls
- Dose / Treatment Duration / Replicates
- Assays used (MTT, apoptosis, etc.)
- Mechanisms / Pathways Studied
- Safety Info (If any effect on normal cells)
- Key Findings
- Limitations / Notes

## For in vivo studies

- Bibliographic details (author, year)
- Drug/Formulation (name, composition, medicine system)
- Animal Species & Strain / Sex / Age / Weight
- Cancer Model Type
- Groups / Controls / Randomization / Blinding
- Dose / Route / Treatment Duration / Replicates
- Outcome Measures
- Mechanisms / Pathways Studied
- Toxicity / Side Effects
- Ethical Compliance
- Key Findings
- Limitations / Notes
